# Photogating in Low Dimensional Photodetectors

**DOI:** 10.1002/advs.201700323

**Published:** 2017-10-04

**Authors:** Hehai Fang, Weida Hu

**Affiliations:** ^1^ State Key Laboratory of Infrared Physics Shanghai Institute of Technical Physics Chinese Academy of Sciences 500 Yutian Road Shanghai 200083 China; ^2^ University of Chinese Academy of Sciences 19 Yuquan Road Beijing 100049 China

**Keywords:** infrared, low dimensional, photodetectors, photogating, phototransistors

## Abstract

Low dimensional materials including quantum dots, nanowires, 2D materials, and so forth have attracted increasing research interests for electronic and optoelectronic devices in recent years. Photogating, which is usually observed in photodetectors based on low dimensional materials and their hybrid structures, is demonstrated to play an important role. Photogating is considered as a way of conductance modulation through photoinduced gate voltage instead of simply and totally attributing it to trap states. This review first focuses on the gain of photogating and reveals the distinction from conventional photoconductive effect. The trap‐ and hybrid‐induced photogating including their origins, formations, and characteristics are subsequently discussed. Then, the recent progress on trap‐ and hybrid‐induced photogating in low dimensional photodetectors is elaborated. Though a high gain bandwidth product as high as 10^9^ Hz is reported in several cases, a trade‐off between gain and bandwidth has to be made for this type of photogating. The general photogating is put forward according to another three reported studies very recently. General photogating may enable simultaneous high gain and high bandwidth, paving the way to explore novel high‐performance photodetectors.

## 1 Introduction

The capability of converting light into electrical signals is essential for modern society. Photodetectors, which have gone through several generations since more than half a century ago, are moving toward high performance, low cost, and small volume, and have reached a high level of maturity in various applications owing to the advances of large‐scale production and integration technologies. Thin‐film materials play a very important role in the development progress of photodetectors. For instance, silicon is widely used and exhibits its irreplaceable role for photodetectors working in the spectrum waveband ranging from 0.4 to 1.1 µm.^1^, ^2^, ^3^ For infrared (IR) photodetectors, typical thin‐film semiconductors with bandgap of 0.1–1 eV like InGaAs, InSb, HgCdTe, and InAs/GaSb type II superlattice dominate the short‐wave infrared (SWIR), mid‐wave infrared, and long‐wave infrared spectrum detection.^4^, ^5^, ^6^, ^7^ The interband or inter‐sub‐band optical transition converts the light into electrical signals. Efficient optical absorption is guaranteed by the enough thickness of thin‐film materials. Generally, these IR detectors work at low temperature, have a very low dark current density, and show excellent performance such as remarkable internal quantum efficiency, fast photoresponse speed, and high detectivity (up to 10^12^ Jones).

Despite the many advantages of thin‐film materials, photodetectors based on low dimensional materials have emerged in recent years and attracted tremendous attention.^8^, ^9^, ^10^, ^11^, ^12^ Here, the “low dimensional materials” means the materials with at least one reduced dimension which is far less than the incident light wavelength. For example, atomically thin 2D materials,^13^ 1D nanowires (NWs)^14^ and nanotubes,^15^ 0D quantum dots (QDs),^16^ nanoplates, and so forth. Due to the limited dimension and large surface–volume ratio, extraordinary light–matter interaction could occur and high optical gain can usually be obtained. NWs own an antenna‐like shape. The absorption cross‐section of NWs at some special wavelengths could be much larger than the cross‐sectional area.^17^ Intuitively, the feature of transparency may make ultrathin 2D materials unsuitable for direct photosensitive alternatives. However, 2D materials could have a strong interaction with light. For instance, monolayer graphene can absorb 2.3% of the incident light from 300 to 2500 nm.^18^ This implies that the optical absorption coefficient reaches ≈10^5^ cm^−1^ over such a wide spectrum. Some ultrathin 2D transition metal dichalcogenides (TMDs) have sharp peaks in the density of states. If these happen to occur near the edges of valance band and conduction band, such as for MoS_2_, WSe_2_, and WS_2_, it will cause an enormously increased optical absorption probability at the photon energy close to the bandgap.^19^, ^20^ The extraordinary light–matter interaction make low dimensional materials full of potential to be used as photodetectors. Overall, limited by the thickness, the light absorption of low dimensionality is still hard to be comparable with thin‐film semiconductors. Nevertheless, the ultrathin thickness enables good electrical tunability through gate voltage and performance improvement by localized fields.^12^

So far, various photodetectors based on low dimensional materials have been reported. Scientists have developed multiple detection mechanisms and designed different device structures to optimize the photodetector performance.^12^, ^20^, ^21^ The photocurrent can be derived from electron–hole (e–h) separation or thermal effect. Due to existence of bandgaps and fast interband optical transition, photoconductors, photo‐field effect transistors (photo‐FETs), and photodiodes are the most studied devices. Especially for 2D materials (graphene, TMDs, boron nitride, black phosphorus, etc.), not only they have a natural broad spectrum distribution of bandgap, but also the bandgap is seriously affected by the thickness or layer numbers.^20^, ^22^, ^23^, ^24^ Thermal detectors widely studied in low dimensional materials are either bolometers or thermopiles, in which the former's resistance is sensitive to temperature and the latter has a Seebeck coefficient difference in its local contacting regions. Generally, thermal detectors have a relatively slow photoresponse speed due to the indirect photoelectric conversion. However, as an exceptional case, graphene has been deeply explored for its photo‐thermoelectric effect (PTE) applications.^9^, ^25^, ^26^, ^27^, ^28^ The hot carriers can be excited ultrafast in graphene and remain at a temperature higher than graphene lattice for many picoseconds.^9^ Therefore, PTE‐based graphene photodetectors can have a high bandwidth.

In this paper, we focus on the low dimensional photodetectors (LDPDs) based on e–h separation and discuss a usually observed phenomenon among these detectors. That is, photogating. Photogating could be simply ascribed to the prolonged excess carrier lifetime induced by defects and impurities or artificial designed hybrid structures.^20^ If one type of the photogenerated carriers is trapped and they have a certain spatial distribution, they can produce an additional electric field like gate voltage to modulate the channel conductance. Most photogating‐dominated LDPDs show high responsivity and limited response speed because of the prolonged excess carrier lifetime. The excess minority carrier lifetime τ is crucial because a trade‐off has to be made between the bandwidth and gain in practice while both are key parameters of a photodetector. Carrier lifetime is closely involved with the type of energy band and defects and impurities in traditional thin‐film semiconductors. For instance, direct bandgap with direct recombination could result in a shorter carrier lifetime than that of indirect bandgap. Impurities and defects can act as the recombination centers and accelerate the recombination of excess carriers. However, due to the large surface‐to‐volume ratio and reduced screening, impurities and defects possibly act as trap centers in low dimensional materials, which greatly prolongs the carrier lifetime rather than recombination centers in most cases. Therefore, many photodetectors dominated by photogating prefer to achieve a considerable photogain, meanwhile, at the cost of response speed.^9^, ^29^, ^30^, ^31^, ^32^

Though photogating has been described as a phenomenon induced by trap states in many literatures, to the best of our knowledge, it has no precise definition.^29^, ^31^, ^33^, ^34^, ^35^, ^36^, ^37^, ^38^, ^39^, ^40^ In this letter, we would like to discuss it in a wider universality. Literally, photogating is a way of modulating the device channel conductance with light‐induced gate field or voltage. Taken in this sense, localized states or hybrid structures cannot be the only alternative to modulate the channel conductance and obtain photogain. Recently, the concept of photovoltage field‐effect transistors was put forward.^40^ This kind of photodetector even enables simultaneous high gain and high bandwidth, which has drawn tremendous attention. However, compared to some hybrid structures enhanced by special photogating,^31^, ^41^, ^42^, ^43^ it shows no much difference in view of the determined equation of gain. Moreover, another interfacial gating enhanced graphene photodetector with high gain–bandwidth product (GBP) has been reported.^44^ The photocurrent in both cases is indirectly determined by a photovoltage. We venture to consider them as general photogating.

Photogating paves the way to design photodetectors with broader detecting spectrum and more excellent performance, and even help to achieve low dimensional room temperature IR photodetectors with remarkable performance for critical applications. In this review, we start from the fundamental concept of photoconductive gain, discuss the characteristics of trap‐ or hybrid‐induced photogating, introduce the recent progress on photogating enhanced LDPDs, reveal the importance of general photogating, and look forward to high‐performance IR photodetectors based on photogating.

## 2 Trap‐ and Hybrid‐Induced Photogating

### 2.1 Gain

Photoconductive gain is widely found in thin‐film photoconductors.^45^ So far, the equation *G* = τ/τ_T_ (τ is the excess minority carrier lifetime, τ_T_ is the carrier transit time) is widely used to estimate the optical gain in photogating enhanced photodetectors.^29^, ^31^, ^39^ Photogating can be regarded as a particular example of photoconductive effect, because both have the same expression of gain and need to work at a bias voltage. There is no clear distinction between them in practice. Assume that a thin‐film photoconductor is uniformly irradiated by incident photons. The net photocurrent could be simply written as *I*_ph_ = *gALe* · *G*, where *g* is the generation rate of excess carriers (cm^−3^ s^−1^), *L* is the channel length, *A* is the cross‐sectional area, *e* is the unit charge, and *G* is the gain. Also, the net photocurrent can be expressed using the photoconductivity by *I*_ph_ = (Δσ · *E*)*A* = (Δ*nμ*_n_ + Δ*pμ*_p_)*eEA*, where ∆*n* and ∆*p* are the excess electron and hole concentrations, *µ*_n_ and *µ*_p_ are the electron and hole mobility, *E* is the electric field intensity provided by bias voltage, respectively. Since Δ*n* = Δ*p*, the gain can be written as *G* = Δ*n*(μ_n_ + μ_p_)*E*/*gL*. Considering the change of excess carrier concentration after illumination, the dynamic process can be described as dΔ*n*/d*t* = *g* − Δ*n*/τ. The derived solution of this above equation using the initial condition of *t* = 0 and Δ*n* = 0, is Δ*n* = *gτ*(1 − *e* ^−*t*/τ^). At the steady state *t* >> τ, hence we get Δ*n* = *gτ*. Then, *G* = τ(μ_n_ + μ_p_)*E*/*L*. If electron is the majority carrier, then the gain can be written as

(1) $$G=(\tau /\tau_{T})(1+\mu_{p}/\mu_{n})\approx \tau /\tau_{T}$$

where τ is excess hole lifetime, τ_T_ = *L*/μ_n_ *E* is the electron transit time. Equation (1) is valid only when μ_p_ << μ_n_ and Δ*n* << *n*_0_, which means a low injection. Because if Δ*n* >> *n*_0_, the excess minority carrier lifetime is no longer a fixed value. It involves the light intensity and time. Moreover ∆*n* would not follow an exponential function. Nevertheless, the equation *G* = τ/τ_T_ is still widely used to estimate optical gain in many reported works when the main photoconductance contribution is from only one type of excess carriers, particularly in some phototransistors or hybrid structures with abundant localized states.

The gain described by Equation (1) can be understood as follows.^46^ During illumination, it takes an average time of τ_T_ for a photogenerated electron to drift through the photoconductor. If the excess minority carrier lifetime is longer than the transit time (τ > τ_T_), once the excess electron reaches the anode, another electron would enter the photoconductor at the cathode immediately to maintain the charge neutrality and drift to the anode terminal. This process repeats until the excess electron recombines with a hole. A period takes an average time of τ and leads to a gain larger than 1. However, if τ < τ_T_, the excess electron would recombine with a hole before finishing a single transit, resulting in a gain lower than 1. Gain lager than 1 without multiple e–h pair generation needs additional power provided by external circuit. Photodiodes working at zero bias voltage cannot produce gain larger than 1.

The gain is available in all photoconductors. However, for a photogating‐dominated low dimensional photoconductor or photo‐FET, it also could exhibit in a very different way. Since one type of the photogenerated carriers is trapped in localized states or hybrid gating layer, they can act as a local gate voltage (Δ*V*_g_) to modulate the channel conductance.^10^, ^29^, ^37^, ^47^, ^48^ The change between the photocurrent and dark current, or the net photocurrent (*I*_ph_) in other words, can be written as

(2) $$I_{\mathrm{ph}}=\frac{\partial I_{d}}{\partial V_{g}}\cdot \Delta V_{g}=g_{m}\Delta V_{g}$$

where *g*_m_ is the transconductance. Because the gain is the ratio between the number of collected carriers by electrodes to produce net photocurrent and the number of excited carriers (see more details in **Table** **1**), it also can be written as

(3) $$G=(g_{m}\Delta V_{g}/e)/(PA\cdot \eta /h\nu )$$

where *P* is the incident light power density (W cm^−2^), *h* is the Planck constant, ν is the light frequency, *PA*/*hv* is equal to the number of photons irradiated on the photodetector in unit time, and η is the quantum efficiency defined as the product of light absorption efficiency and charge transfer efficiency. In some cases η is ignored, then the gain has the same significance as external quantum efficiency. See the definitions of more parameters usually used in photoconductors and phototransistors in Table 1. Note that the sign of transconductance depends on the majority carrier polarity in channel whereas Δ*V*_g_ is closely related to the type of localized trap states or the band alignment of the heterojunction. For instance, if the majority carrier is the electron and the localized states mainly trap holes (hole‐trap states), then it would be given *g*_m_ > 0 and Δ*V*_g_ > 0. Hence a positive photocurrent would be obtained. Nevertheless, both *g*_m_ and Δ*V*_g_ can be either positive or negative. Therefore, the photocurrent can also be a negative value, which is seldom observed in thin‐film photoconductors. In addition, the gain described by Equation (3) indicates a gate voltage‐dependent relation and a maximum responsivity achieved at the maximum transconductance around the threshold voltage.

**Table 1:** Definitions of several key parameters in photoconductors and phototransistors

Parameters	Definitions
Responsivity (*R*)	The net photocurrent divided by the incident light power: *I*_ph_/*P*_in_, in units of A W^−1^ (or V W^−1^ if a photovoltage is measured).
Quantum efficiency (η)	Defined as the product of light absorption efficiency and charge transfer efficiency. It equals the ratio between the number of excited electron–hole pairs and the number of incident photons.
Gain (*G*)	The number of photogenerated electron–hole (e–h) pairs collected by the electrodes to produce the net photocurrent divided by the number of photoexcited e–h pairs, written as *G* = (*I*_ph_/*e*)/(*φAη*), where φ is the photon flux in units of cm^−2^ s^−1^, *A* is the effective absorption area. Gain could be estimated by the ratio between excess carrier lifetime and carrier transit time: τ/τ_T_. In some cases, η is ignored, then gain has the same meaning as external quantum efficiency.
3 dB bandwidth	The modulation frequency *f*_T_ of incident light when the responsivity (or gain) decreases 3 dB (≈0.707 of the original value). When the modulation frequency is much lower than *f*_T_, it can be considered that the responsivity is independent of modulation frequency.
Response time (τ)	The time costed for the current to arrive at a stable level after receiving (or removing) photon flux, also named as rise time (or decay time). Response time can be approximately equal to the average excess carrier lifetime. In many cases, the response time of a photoconductor could be estimated by τ = 1/2*πf*_T_.
Gain–bandwidth product (GBP)	The product of gain and bandwidth. It equals to the modulation frequency of light when the gain decreases to 1. The GBP can be roughly estimated by *g*_0_/τ, where *g*_0_ is the measured gain without light modulation.

### 2.2 Characteristics

#### 2.2.1 Gain and Response Time

**Figure 1:**
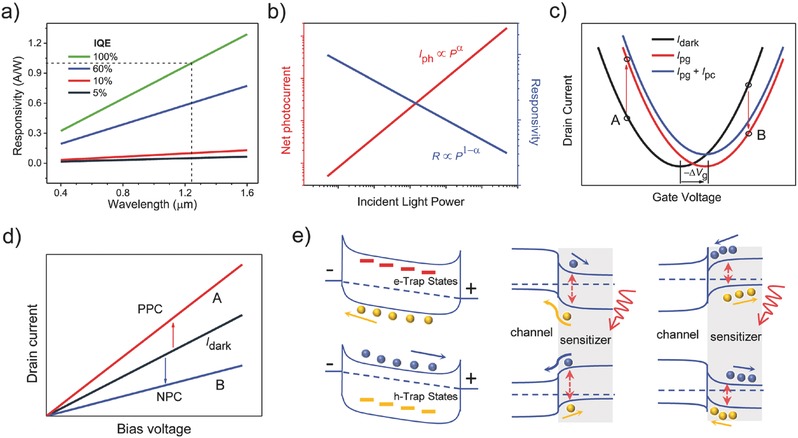
Identification of photogating. a) Wavelength dependence of responsivity at different internal quantum efficiency when the gain is ignored. b) Power law: nonlinear light power dependence of net photocurrent and responsivity (0 < α < 1). c) *I*_ds_–*V*_g_ trace shift after illumination. Dark line, red line, and blue line represent the dark current, photocurrent of photogating effect, photocurrent of photogating and photoconductive effect, respectively. Δ*V*_g_ is the photoinduced local gate voltage as mentioned in the main text. Because the trace shift direction and photovoltage have an opposite sign, here we use −Δ*V*_g_ to denote the trace shift direction. d) *I*_ds_–*V*_d_ curves of points A and B in panel (c) in dark and under illumination. e) Schematic diagrams of band alignments of trap‐ and hybrid‐induced photogating. Left panel: p‐type material with electron‐trap states (top part) and n‐type material with hole‐trap states (bottom part); Middle panel: e–h pairs are generated in the sensitizer and electrons remain in the gating layer whereas holes get into the channel (top part), or holes remain in the gating layer whereas electrons get into the channel (bottom part); Right panel: e–h pairs are generated in the sensitizer and electrons are accumulated at the interface (top part), or holes are accumulated at the interface (bottom part).

The responsivity of fully absorbing incident light with photon energy of 1 eV (λ = 1.24 µm) and quantum efficiency (QE, see definition in Table 1) of 100% is 1 A W^−1^. **Figure** **1**a depicts the relation between responsivity and wavelength at different QE without gain, *R* = η · *eλ*/*hc*. Take photo‐FETs based on bare 2D materials as examples. Ultrathin 2D materials fail to show sufficient optical absorption, and the QE is poor due to the strong exciton effect in some monolayers. Suppose that a few‐layer 2D material absorbs 10% of the incident laser with λ = 612 nm and the charge transfer efficiency reaches 50% (therefore the QE is 5%, this is rough but reasonable^9^), the responsivity would be 25 mA W^−1^, regardless of the gain. However, a number of reported 2D material photodetectors show much higher responsivity, as shown in **Table** **2**, revealing an obvious gain. Gain produced by prolonged carrier lifetime limits the response speed and bandwidth. Thin‐film photoconductors and photodiodes could have a bandwidth of more than 1 GHz, while to our best knowledge so far, no reported LDPDs with obvious photogating has a bandwidth even over 1 MHz. Localized states greatly prolong the releasing process of trapped carriers for recombination. Guo et al. has reported a mid‐infrared (MIR) black phosphorus (BP) photodetector with shallow trap states,^29^ while carrier lifetime still reaches as long as ≈0.13 ms. Liu et al. have achieved a carbon nanotube/graphene hybrid photodetector with high GBP with a response time limited to 0.1 ms.^39^

**Table 2:** Figures‐of‐merit in typical low dimensional photodetectors

System	Material	Responsivity [A W^−1^]	Gain	Response time [s]	Detection range [µm]	GBPa)	Ref.
Nanowires	ZnO	–	2 × 10^8^	10	UV	6 × 10^9^	67
	Zn_3_P_2_	–	470	2 × 10^−5^	Visible	≈10^7^	55
	InAs	–	−10^5^	≈10^−2^	Visible–NIR	≈10^7^	38
	InAs	−3 × 10^4^	−7.5 × 10^4^	<5 × 10^−3^	Visible	≈10^7^	65
	InP	2.8 × 10^5^	4.2 × 10^5^	10^−1^	Visible–NIR	≈10^6^	17
	CdS	2.6 × 10^5^	8.6 × 10^5^	10^−1^	UV	≈10^7^	56
	InAs	40b)	–	6 × 10^−5^	NIR–MIR	≈10^5^	75
	InAs	5.3 × 10^3^	–	<10^−2^	Visible–NIR	≈10^5^	72
	GaAs	–	2 × 10^4^	–	Visible	–	68
2D materials	Graphene	5 × 10^−4^	–	≈2.5 × 10^−11^	1.55	≈10^7^	104
	Graphene	8.61	–	>10	Visible–MIR	–	57
	MoS_2_	2200	5000	–	Visible	–	58
	MoS_2_	880	–	4	Visible	≈10^2^	105
	InSe	12.3	–	5 × 10^−2^	Visible	≈10^2^	106
	WSe_2_	–	10^5^	2.3 × 10^−2^	Visible	≈10^6^	107
	In_2_Se_3_	10^5^	–	9	Visible–NIR	≈10^4^	47
	MoS_2_	10–10^4^	–	10^−2^–10	Visible	≈10^3^	48
	BP	82	≈10^4^	10^−4^	MIR	≈10^8^	29
	MoTe_2_	10^−2^	–	10^−3^	Visible–NIR	≈10^1^	59
	ReS_2_	8 × 10^4^	–	≈10^2^	Visible	≈10^3^	102
	BP	10^6^	–	5 × 10^−3^	0.4–0.9	≈10^7^	60
	BP	0.657	–	≈3 × 10^−10^	NIR	≈10^9^	83
Nanosheets/nanoplates	GaS	19.2	93.7	<3 × 10^−2^	UV	≈10^3^	108
	GaSe	3.5	5.3	0.1	0.8	≈10^1^	109
	Bi_2_S_3_	4.4	8.6	10^−5^	Visible–NIR	≈10^5^	110
	SnS_2_	8.8 × 10^−3^	2.4 × 10^−2^	5 × 10^−6^	Visible	≈10^4^	111
Low dimensional hybrid structures	Graphene/QDs	10^7^	10^8^	10^−2^	Visible–NIR	10^10^	31
	Graphene/QDs	10^7^	–	0.26	NIR	≈10^7^	41
	Graphene/MoS_2_	10^10^ c)	10^10^	1	Visible	≈10^10^	32
	Graphene/Ti_2_O_5_/graphene	>1	–	–	NIR–MIR	–	91
	Graphene/MoS_2_	1.2 × 10^7^	≈10^8^	–	Visible	–	88
	Perovskite/graphene	180	500	≈10^−1^	Visible	≈10^3^	89
	Graphene/carbon nanotube	>100	10^5^	10^−4^	Visible–NIR	10^9^	39
	QDs/InGaZnO	≈10^6^	≈10^6^	≈10^0^	Visible–NIR	≈10^6^	42
	QDs/WSe_2_	2 × 10^5^	–	10^−2^–10^0^	NIR	<10^7^	43
	QDs/Si	–	10^4^	10^−5^	NIR	10^9^	40
	Graphene/SiO_2_/light doped Si	1000	–	4 × 10^−7^	0.514	≈10^9^	44

^a)^ “≈” represents a roughly estimated GBP through *g*/τ

^b)^ This performance is achieved at 77 K

^c)^ Achieved at 130 K.

Gain and bandwidth are competing owing to limitation by excess carrier lifetime. The maximum bandwidth without decay of gain can be roughly estimated by 1/τ whereas gain is proportional to τ/τ_T_. Hence, the GBP related to the transit time τ_T_ would be a constant once the applied bias voltage, channel length, and carrier mobility are determined. Gain in some photo‐FETs can vary greatly at different gate voltages due to the gate‐controlled number of trap states. However, the law that a larger responsivity suggests a lower response speed cannot be broken. The maximum responsivity and the fastest response time obtained at different gate voltages cannot precisely reflect the performance of a photodetector. The GBPs of some LDPDs have been listed in Table 2. Note that these data are not absolutely accurate. Many literatures did not experimentally measure the GBP, and the time response in some work was divided into a fast and a slow rising process, while the stable photocurrent and only the fast component were used for estimation.

#### 2.2.2 Nonlinear Power Dependence

Some traditional thin‐film photodetectors and novel high‐performance photodetectors have a wide linear dynamic range,^49^, ^50^, ^51^, ^52^ which means that the photocurrent shows a linear relation with the incident light power before saturated absorption. Hence, there is *I*_ph_ ∝ *P* ^α^, where the power exponent α is equal to 1 or very close to 1 in practice. However, a nonunity exponent of 0 < α < 1 is often found in LDPDs,^17^, ^31^, ^38^, ^41^, ^43^, ^47^, ^53^, ^54^, ^55^, ^56^, ^57^, ^58^, ^59^, ^60^, ^61^, ^98^ as a result of the complex process of carrier generation, trapping, and recombination within semiconductors.^54^ Since the responsivity can be derived from *R* = *I*_ph_/*P*, there is *R* ∝ *P* ^−(1 − α)^, as shown in Figure 1b. This relation is also available to gain. Gain decreases with increasing light power partly due to the gradually filled trap states. Once all the trap states are fully filled at a certain intensity of light power, a stronger light power would excite more free carriers that cannot be trapped, resulting in a decreasing average carrier lifetime. Therefore, the gain is reduced. It is conceivable that the responsivity may not change in a range of very weak power density when the trap states are sufficient.^31^, ^32^ Responsivity reported in different works may be not appropriate to be directly used for comparing the photosensitivity, because they could be measured at different power densities. Especially for some photodetectors with α close to zero (*R* ≈ *P* ^−1^),^60^ an incident power density of two orders of magnitude lower implies a responsivity of two orders of magnitude higher. Though no uniform standard on power density used for calculating the responsivity has been applied, it is found that a power density of several orders of magnitude lower than 1 mW cm^−2^ is commonly used to check the sensitivity of photodetectors. Note that the nonlinear or power‐law dependence of photocurrent on incident light power can be a characteristic of photogating dominated LDPDs, while it is not necessary that all the nonlinear or power‐law relations in LDPDs are attributed to photogating.

#### 2.2.3 I_ds_–V_g_ Trace Shift and Negative Photoconductance

Gain depicted by Equation (2) reveals the importance of the horizontal shift of *I*_ds_–*V*_g_ trace (simply shown in Figure 1c) in photogating‐dominated LDPDs. According to most reported work, three main cases that can cause *I*_ds_–*V*_g_ trace shift are summarized in Figure 1e. For nonhybrid (or bare material) structures, the specific spatial distribution of surface or interface trap states is the main source (see the left panel in Figure 1e). During illumination, these trap states can trap one type of photogenerated carriers and collectively generate a gate electric field to modulate the channel conductance. Because the filling of trap states is involved with the Femi level, n‐type channel mainly has hole‐trap states and p‐type channel mainly has electron‐trap states. For hybrid structures, the charge exchange through the heterointerface (middle panel in Figure 1e) or charge accumulation at the interface (right panel in Figure 1e) can cause *I*_ds_–*V*_g_ trace shift. Here, we only consider the case that the photosensitive material (sensitizer) is the gating layer instead of the channel material. This trace shift largely depends on the energy band alignment in the heterostructure. The built‐in field at the interface or band bending near the surface of the sensitizer enables efficient e–h separation, and the photocarriers concentrated in the nonchannel layer will produce an electric field to modulate the channel conductance.

Photogating may lead to negative photoconductance (NPC). Take the *I*_ds_–*V*_g_ trace shift in Figure 1c as an example. The transfer characteristic trace shifts to the positive direction after illumination suggests a negative gating induced by trapped or hybrid‐concentrated electrons (Δ*V*_g_ < 0). The photoresponse behaviors at points A and B (see Figure 1c) are totally different. When the device is working at point A, the majority carrier in dark is hole (*g*_m_ < 0), hence the photocurrent described by Equation (2) is positive. While for the working point B, the majority carrier in dark is electron, indicating a positive *g*_m_ and a negative photocurrent. Figure 1d shows the *I*_ds_–*V*_g_ curves for the working points A and B in dark and under illumination. The sign of photoconductance is essentially determined by the change of the number of majority carrier in channel (suppose the contact is always good). If the type of the photogenerated carriers injected into the channel is same to that of the majority carrier of channel material, or the type of the photogenerated carriers left in the gating layer or trap states is opposite to that of the majority carrier of channel material, then a positive photoconductance (PPC) will be obtained. Otherwise, a NPC could be measured.

In fact, the horizontal shift of *I*_ds_–*V*_g_ trace depicted in Figure 1c is very ideal and cannot be so uniform in practice. First, it should be noted that the photoinduced local gate voltage Δ*V*_g_ has a big relation with the applied gate voltage. For a bare channel, the electron‐trap states would be gradually filled by electrons with the rise of Fermi level and hole‐trap states would be gradually filled by holes with the fall of Fermi level tuned by the gate voltage. Therefore, the photoinduced Δ*V*_g_ determined by the number of available trap states would change under different gate voltages. For hybrid structures, the gate voltage is able to tune the heteroband alignment and affect the e–h separation efficiency, thus also leading to a gate‐voltage dependent Δ*V*_g_. Hence, the *I*_ds_–*V*_g_ trace shift should not be so uniform in practice. Besides, due to limited trap states and incomplete trapping of photoexcited carriers in bare channel, the *I*_ds_–*V*_g_ trace may have a vertical shift, as shown in Figure 1c (see the blue curve), indicating a photoconductive photocurrent component.

Here, we want to further emphasize that the NPC is more likely to be discovered in hybrid structures rather than LDPDs based on bare semiconductor. Because, the NPC needs the coexistence of many majority carriers and majority‐carrier‐trap states. Generally, most majority‐carrier‐trap states have been filled by the majority carriers before illumination, making them hard to generate an obvious photovoltage. So far, the NPC has been observed in several nonhybrid nanostructures such as p‐type carbon nanotubes^62^ and Bi‐doped ZnSe NWs,^63^ n‐type InN thin films,^64^ and InAs NWs.^38^, ^65^ However, the NPC in these p‐type systems is attributed to the photoassisted oxygen desorption which releases electrons and reduces the hole concentration.^62^, ^63^ In n‐type InN films, light‐enhanced carrier scattering reduces the mobility and causes the NPC.^64^ Only the NPC in InAs NWs is caused by photogating. However, the core/shell InAs NW with inhomogeneous composition or native oxide layer,^38^ or rather, the model that the energy level of trap states is 0.5 eV above the conduction band isolated by a barrier height of 0.6 eV,^65^ can also be regarded as a hybrid structure. Therefore, The NPC in hybrid structures is almost the evidence of photogating.

### 2.3 Recent Progress of Photogating in LDPDs

#### 2.3.1 NW Photodetectors

**Figure 2:**
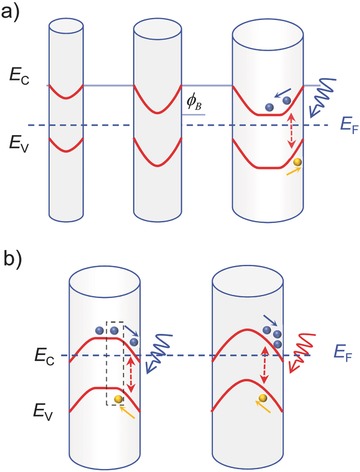
Nanowire (NW) surface band bending and its impact on photoconductance. a) Diameter dependence of conduction and valance band shapes in an n‐type NW with hole‐trap surface states. Left panel: small surface band bending and full depletion of NW at small diameters; Middle panel: surface band bending and full depletion of NW at critical diameter; Right panel: the NW is not fully depleted for larger diameters. When under illumination, photogenerated electrons and holes are separated under built‐in field. φ_B_ is the recombination barrier. Reproduced with permission.^69^ Copyright 2005, American Chemical Society. b) Left panel: photogating‐induced negative photoconductance in InAs NWs. The blue light trace indicates an illumination with much higher photon energy than the bandgap of InAs, and the black dashed box represents the recombination between the photogenerated holes and free electrons. Right panel: because of the accumulated electrons on the NW surface (this could be achieved by high‐energy photon illumination at low temperature), the InAs NW core is nearly fully depleted. Subsequently, an infrared light (with lower photon energy as depicted in red light trace) could cause the positive photoconductance.

Semiconductor NWs enable highly efficient photon capture and process them as electrical outputs.^8^ The conversion of NWs from insulating to conducting state when receiving photon flux sparks great interest to study optical switches and photodetectors based on NWs, as shown in Table 2.^54^, ^56^, ^66^, ^67^, ^68^ Because of the large surface‐to‐volume ratio, surface states play a significant role in NW sensors. For NW photodetectors, the surface states and surface Fermi level pining induced band bending has a great impact on the separation and recombination of excess carriers.^69^ Both the number of surface states and NW diameter matter a lot, as shown in **Figure** **2**a. Suppose that the surface states mainly trap holes and an upward band bending occurs. When the diameter is small enough, the NW is fully depleted and the band bending is minimal. There is no effective barrier to prevent the recombination of photogenerated electrons and holes. Thus, the gain is limited and the response speed is guaranteed. When the diameter reaches a critical value, the recombination barrier φ_B_ induced by the increasing band bending works, leading to a prolonged carrier lifetime. The right panel in Figure 2a depicts an n‐type NW with larger diameter and abundant surface hole‐trap states. When under illumination, photoexcited electrons move to the NW core whereas holes move to the surface. The spatial isolation of excess holes and electrons enables an additional positive gate voltage produced by the concentrated holes on NW surface modulating the core conductance. This is a typical photogating. When the light is switched off, the excess electrons and holes should overcome a barrier of φ_B_ to recombine with each other, thus making it take a long time for the photocurrent to drop to the original level. In some extreme cases such as some ZnO NWs, the photocurrent is unable to recover to the level of initial dark current after removing the light source, indicating a persistent photoconductance.^70^, ^71^ In addition to the surface defects, the adsorption of gas molecules in atmosphere may cause surface states, especially for oxygen with strong electronegativity.^54^, ^67^, ^70^, ^72^, ^73^

**Figure 3:**
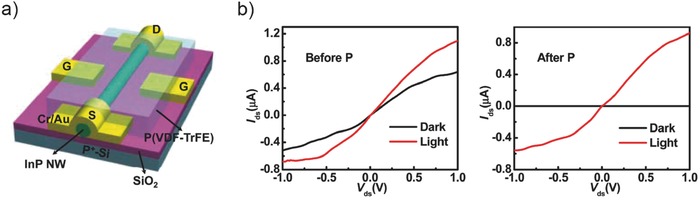
Ferroelectric field enhanced NIR InP nanowire photodetector. a) Schematic diagram of the device structure. b) *I*_ds_–*V*_ds_ curves in dark and under illumination before ferroelectric polarization (left panel) and after negative polarization (right panel). Reproduced with permission.^17^ Copyright 2016, American Chemical Society.

High optical gain or responsivity has been widely reported in single NW photodetectors such as InAs,^72^ InP,^17^ CdS,^56^ ZnO,^54^, ^67^ GaAs,^74^ Zn_3_P_2_,^55^ etc. In addition to the long excess carrier lifetime, short channel and good quality of NW crystal would reduce the transit time and improve the gain. Early in 2007, Wang and co‐workers realized a UV ZnO NW photodetector with high gain more than 10^8^.^67^ The large recombination barrier induced by the oxygen adsorption assisted surface band bending is the inner source of the ultrahigh gain. Despite the slow relaxation time of ≈10 s, a GBP as high as 6 × 10^9^ Hz was obtained, which is comparable to traditional thin‐film photodetectors. In 2016, Zheng et al. demonstrated a side‐gate ferroelectric field enhanced InP near‐infrared (NIR) NW photodetector (see **Figure** **3**a) with an ultralow dark current (approximately in picoamperes) and an ultrahigh gain of ≈10^5^.^17^ The large electric field produced by negative polarization state of poly(vinylidene fluoride‐trifluoroethylene) (P(VDF‐TrFE)) ferroelectric polymer can keep the InP NW at a full depleted state. However, the authors failed to explain the gain mechanism and why the net photocurrent after polarization is even larger than before (see Figure 3b). Here, we provide a feasible explanation and attribute it to photogating. Because the strong ferroelectric field applied on the NW surface would repel the near‐surface electrons and cause sharp upward surface band bending as shown in Figure 2a (middle panel), the photoexcited electrons and holes could be spatially isolated and have a longer lifetime, thus leading to a high gain. In fact, the net photocurrent with close magnitude before negative polarization also implies a large gain. While, the carrier lifetime after a negative polarization could be longer owing to the wider depletion region and higher recombination barrier. Therefore, the net photocurrent could be larger after negative polarization. This explanation is also available to the CdS NW photodetectors enhanced by ferroelectric field.^56^

**Figure 4:**
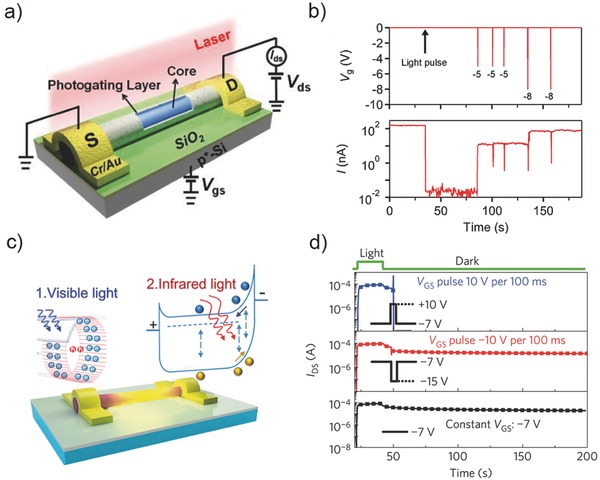
a) Schematic diagram of photogating dominated core/shell like InAs nanowire (NW) phototransistor. Reproduced with permission.^38^ b) Gate voltage pulse controlled drain current in low temperature InAs NW memory. Reproduced with permission.^65^ Copyright 2015, American Chemical Society. c) Schematic diagram of visible light‐assisted infrared InAs NW photodetector. Reproduced with permission.^75^ Copyright 2016, American Chemical Society. d) Gate pulse controlled recover process of drain current after illumination in oxide thin‐film transistor. Reproduced with permission.^71^ Copyright 2012, Nature Publishing Group.

Though most reported photogating enhanced NW photodetectors show large positive photogain, the NPC has also been observed.^38^, ^65^, ^75^ As discussed in Section 2.2.3, InAs NWs with electrons as majority carrier and electron‐trap states could result in NPC. In 2014, Guo et al. observed this phenomenon and attributed it to photogating (see **Figure** **4**a).^38^ Photoresponse based on majority carrier transport exhibits a large negative photogain as high as −10^5^. Yang et al. explored the quenched detrapping process at low temperature and put forward the concept of low temperature memory based on single InAs NW (Figure 4b).^65^ Subsequently, our group took advantage of the long kept NPC induced by high energy photon flux and designed visible‐light‐assisted single InAs NW photodetectors with wide detection range from NIR to MIR and short response time of less than 80 µs (Figure 4c).^75^ These study results also could be illustrated by surface band bending, as shown in Figure 2b. InAs has been found to have accumulated electrons on surface and causes downward surface band bending.^76^, ^77^, ^78^ For InAs NW with limited diameter, when under photoexcitation, photogenerated electrons move to the surface and are trapped whereas holes are left in the core and recombine with free electrons, leading to the NPC. Note that the detrapping process is thermal assisted. A photon flux with energy of much higher than the InAs bandgap would cause more electrons trapped by surface states at low temperature, thus leading to a near full‐depleted NW core (see Figure 2b, right panel). An infrared irradiation at this time would generate free holes in the NW core and increase the conductance, thus successfully utilizing the NPC to achieve PPC. Due to the limited hole mobility and response region (Schottky junction), the gain in the visible light‐assisted InAs NW photodetectors is also restricted. The reported responsivity in MIR range is 0.6–40 A W^−1^.^75^

Surface state induced photogating in NW photodetectors improves the gain, while it reduces the response speed. Applying gate voltage pulse is an electrical method to tune the filling state of trap states and accelerate the detrapping process. For the persistent photoconductance in ZnO NWs or thin films, the trapped holes which are hard to be recombined is the cause. Increasing the probing current^70^ or applying a positive gate voltage^71^ is effective to promote the recombination probability by electron injection. Jeon et al. developed an active‐matrix photosensor array based on amorphous oxide semiconductor photo‐thin‐film transistor and achieved high‐frame imaging with the assistance of nanosecond gate pulse (see Figure 4d).^71^ On the contrary, trapped electrons can be recombined by negative gate voltage pulse induced hole injection in the case of InAs NW photodetectors.^65^, ^75^

#### 2.3.2 Bare 2D Material Photodetectors

**Figure 5:**
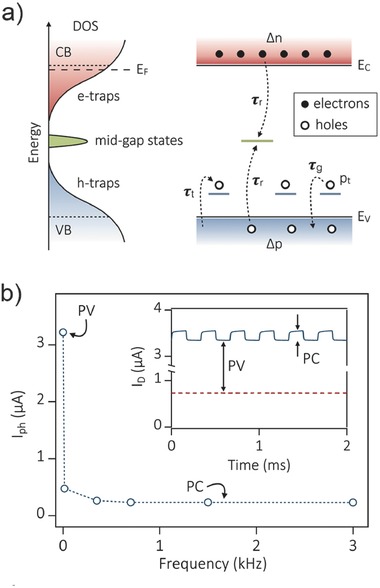
Photogating and photoconductive effect in monolayer MoS_2_ photodetector. a) Schematic diagram of density‐of‐states (DOS) and simplified energy band diagram with features of charge trapping model. b) Photocurrent versus optical modulation frequency. The slow component which quenches at high frequency is dominated by photogating. Reproduced with permission.^37^ Copyright 2014, American Chemical Society.

**Figure 6:**
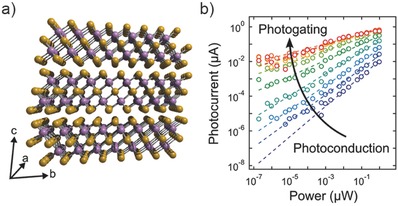
a) Crystal structure of In2Se3. b) Photocurrent versus incident power at different gate voltages. Reproduced with permission.^47^ Copyright 2015, American Chemical Society.

Photogating in bare 2D material photodetectors is caused by trap states. As a typical semiconductor of TMDs, MoS_2_ photodetectors have displayed a large variation in performance.^37^, ^48^, ^57^, ^58^, ^79^ Structure defects or disorder in MoS_2_ can result in band tail states in the conduction and valance band.^80^ Furchi et al. studied the photoconductivity mechanisms in atomically thin MoS_2_ and confirmed the coexistence of both photogating effect (also named as photovoltage effect in their paper) and photoconductive effect.^37^ Photogating effect is caused by the photogenerated carriers transferred to the MoS_2_/SiO_2_ interface or nearby molecules, which can act as an additional gate voltage and result in threshold shift. While, the excess carriers trapped by the band tail states (see **Figure** **5**a) contribute to the photoconductive effect. Compared to the latter, the photocurrent component of photogating is larger in magnitude but slower in speed, as shown in Figure 5b. A high‐frequency oscillating irradiation can extract the photoconductive component through phase lock‐in technique. However, they have no obvious distinctions except the time scale. Note that the photogating component determined by Equation (2) greatly depends on the back‐gate voltage *V*_g_. However, the photoconductive component is not sensitive to *V*_g_, though it drops a little with decreasing *V*_g_ due to the downward shifting Fermi level induced emptied states near the conduction band edge. The similar phenomenon has also been observed by Kufer and Konstantatos.^48^ The responsivity and temporal response of their MoS_2_ device can be tuned over several orders of magnitude by controlling the gate voltage. When applied a strong negative *V*_g_, this detector exhibits a faster speed on the order of millisecond and higher detectivity of more than 10^11^ Jones. Island et al. have shown us a more intuitive conclusion in their study of In_2_Se_3_ photodetectors, as shown in **Figure** **6**.^47^ Photocurrent versus laser power at different back‐gate voltages depicts an evident power law as discussed in Section 2.2.2. For the large negative back‐gate voltage, the power exponent α is ≈1, indicating a linear relation between the net photocurrent and incident power. At this condition, photoconductive effect dominates the photoconductance. With increasing gate voltage from −40 to 40 V, α decreases to ≈0.3 and photogating becomes the dominant mechanism. A high responsivity of ≈10^5^ A W^−1^ has been achieved with the assistance of photogating.

**Figure 7:**
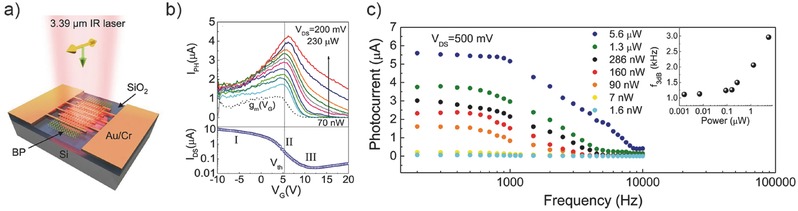
Photogating enhanced mid‐infrared black phosphorus photodetector. a) Schematic view of the device structure. b) Maximum photocurrent under various incident powers achieved at around the maximum transconductance. c) Photocurrent amplitude versus optical modulation frequency under various incident powers. Reproduced with permission.^29^ Copyright 2016, American Chemical Society.

Huang et al. realized a visible‐NIR BP photodetector with ultrahigh responsivity of 10^6^ A W^−1^ by reducing the carrier transit time.^60^ The exponent α of power law is even close to 0 in a wide temperature range, indicating an impressive function of photogating. Furthermore, the response time measured at a strong illumination intensity reaches several milliseconds, and a remarkable GBP of ≈10^7^ Hz could be estimated at the same incident power. Yet, this work has not extracted the full potential of BP, because BP covers a wide electromagnetic spectrum and is a good alternative for polarization‐sensitive infrared detection.^81^, ^82^ Waveguide‐integrated BP photodetector has been reported to exhibit a bandwidth as high as 3 GHz at the important telecom wavelength.^83^ Guo et al. achieved a MIR BP photodetector with high gain, which was proved to be attributed to the photogating effect.^29^ The photocurrent of this BP photodetector (see the device schematic in **Figure** **7**a) peaks at the maximum transconductance point around the threshold voltage as shown in Figure 7b, which is consistent to Equation (2), implying the dominant position of photogating. This conclusion is valid especially when the incident power is low. Under higher light intensity, with more photogenerated trapped carriers, the maximum point of the net photocurrent horizontally shifts a little to the positive direction, and with gradually filling trap states, more free carriers contribute to the conductance even at off state. Enhanced by the photogating mechanism, the gain is as high as 10^4^ whereas the bandwidth reaches 1.2 kHz (Figure 7c). Owing to the low dark current, this photodetector is able to detect a picowatt MIR light at room temperature.

#### 2.3.3 Hybrid Structures Based on QDs and 2D Materials

Colloidal QDs are favored to be integrated with other 2D materials such as graphene, MoS_2_, and WSe_2_ for fabricating hybrid photodetectors.^31^, ^40^, ^41^, ^43^, ^84^, ^85^, ^86^, ^87^ Due to the tunable bandgap induced by confinement effect, PbS QDs have been widely used for photodetection. By controlling the size of QDs during synthesis, the exciton peak can be tuned over a wide spectrum ranging from 500 to 2100 nm.^85^ However, pure QD IR photodetectors suffer from the low carrier mobility, greatly impeding the detector performance. Integrating QDs with 2D materials has several advantages. First, 2D materials fail to show strong optical absorption, while QDs with thicker thickness can efficiently utilize the light. Second, 2D materials acting as the channel can avoid the problem of very low mobility. Third, the broadband absorption of QDs can compensate the limited response waveband of some 2D materials. Moreover, spin coating technique of colloidal QDs can be used for future array‐based imaging system. Here, we separately write this section to address the importance of hybrid structures integrating 2D materials with QDs.

**Figure 8:**
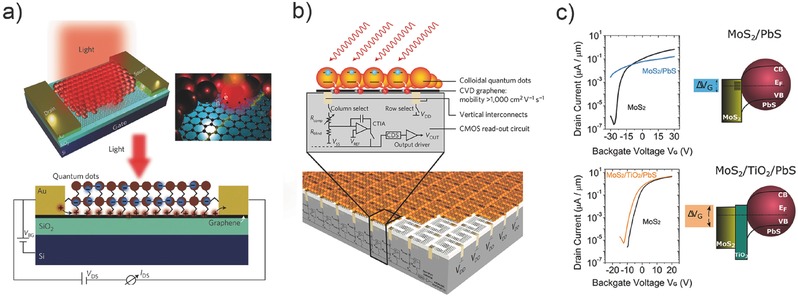
QD/2D material hybrid photodetectors. a) Schematic view of hybrid graphene–quantum dot phototransistors. Reproduced with permission.^31^ Copyright 2012, Nature Publishing Group. b) Side view of the photogating enhanced graphene/QD imaging sensor and the underlying read‐out circuit. Reproduced with permission.^87^ Copyright 2017, Nature Publishing Group. c) Transfer curves of MoS_2_/PbS QDs device before and after inserting a TiO_2_ insulating layer between QDs and MoS_2_. Reproduced with permission.^86^ Copyright 2016, American Chemical Society.

Early in 2012, Konstantatos et al. demonstrated a QD/graphene hybrid phototransistor with ultrahigh gain of 10^8^, as shown in **Figure** **8**a.^31^ The deposition of QDs shifts the Dirac point of graphene from 120 to 50 V, implying a hole transfer from graphene to QDs, thus a built‐in field is formed at the interface. Under photoexcitation, electron–hole pairs are generated in QDs. Holes are transferred to the graphene, whereas electrons are trapped in the QD layer and modulate the conductance of graphene. Owing to the prolonged excess electron lifetime in graphene and high mobility, an ultrahigh responsivity of ≈10^7^A W^−1^ was obtained at a weak incident power of 50 fW. The GBP of this hybrid photodetector reaches 10^9^ Hz, which is comparable to III–V phototransistors grown by harsh and expensive molecular beam epitaxy (MBE). In the same year, Sun et al. reported a chemical vapor deposition (CVD) graphene/QD photodetector with responsivity of 10^7^ A W^−1^.^41^ Interestingly, this device was fabricated on flexible plastic substrates, revealing the bendability and wearability. Very recently, based on the photogating enhanced mechanism, Goossens et al. achieved an integrated QD–graphene–complementary metal‐oxide‐semiconductor (CMOS) image sensor.^87^ To our best knowledge, this is the first time to incorporate large‐area 2D materials with silicon readout circuit and realize a NIR–SWIR digital camera with photodetection pixels of a 388 × 288 array. This result depicts the essential significance of photogating for future practical applications.

PbS QDs have also been used to incorporate with other 2D materials and even metal oxide.^42^, ^43^, ^84^, ^86^ Owing to the light absorption of QDs, many devices respond in the NIR spectrum. For instance, the reported QDs/MoS_2_ photodetector has broken the bandgap limitation of MoS_2_ and extended the detection wavelength to 1550 nm.^84^ The high sensitivity for weak light detection is the common feature of this type of photodetectors (see Table 2). The interface engineering between MoS_2_ and PbS QDs has been studied by Kufer et al.^86^ It was found that after depositing the QDs, the MoS_2_ FET showed a much higher dark current at the off state. This phenomena has also been observed according to the experimental data in another work of Kufer et al. (see Figure 1d in ref. 84). Inserting a thin insulating layer between MoS_2_ and QDs can reduce the dark current at the off state and raise several orders of magnitude of the on/off ratio, as shown in Figure 8c. Furthermore, the response speed has also been improved.

The on/off ratio of a photo‐FET is essential for photogating. An efficient photogating should be able to tune the transistor between a distinct on/off state. However, high mobility and low dark current should be made a trade‐off. For instance, graphene has a high mobility while the dark current is large. On contrast, the dark current of MoS_2_ can be tuned to a very low level while the mobility is low, limiting the gain. As pointed out by Kufer and Konstantatos, the best performance of a 0D/2D photo‐FET depends on the operational regime, where the dark current, gain, and quantum efficiency are optimized.^85^ The incorporation of thin channel which is easier to be depleted and suitable sensitizer with compensatory optical absorption and highly efficient e–h separation would be a very good choice.

#### 2.3.4 Hybrid Structures Using Graphene as Channel

**Figure 9:**
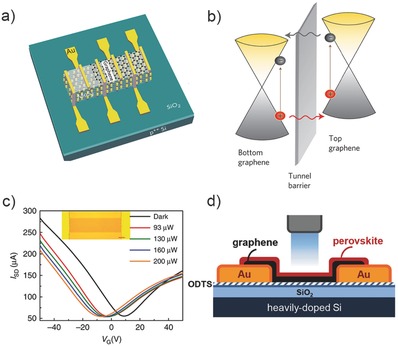
Graphene‐based hybrid photodetectors. a) Device structure of hybrid graphene–MoS_2_ photodetector. Reproduced with permission.^32^ Copyright 2013, Nature Publishing Group. b) Working principle of sandwiched graphene/Ta_2_O_5_/graphene hybrid MIR photodetector. Reproduced with permission.^91^ Copyright 2014, Nature Publishing Group. c) *I*_ds_–*V*_g_ trace shifts of hybrid graphene/carbon nanotube photodetector under different incident light powers. Reproduced with permission.^39^ Copyright 2015, Nature Publishing Group. d) Schematic diagram of the hybrid graphene/perovskite photodetector. Reproduced with permission.^89^

Gain determined by τ/τ_T_ is greatly dependent on the carrier transit time, which can be reduced with shorter channel length, larger bias voltage, and higher carrier mobility. To achieve a high performance, graphene is preferred to be used as the channel of a hybrid structure due to its high mobility.^32^, ^39^, ^88^, ^89^, ^90^, ^91^ In the year of 2013, Roy et al. incorporated graphene with MoS_2_ wherein graphene acts as the channel and MoS_2_ acts as the gating layer, as shown in **Figure** **9**a.^32^ Negative back‐gate voltage induced band alignment at the interface enables photoexcited electrons transfer to graphene whereas holes remain in MoS_2_. The responsivity of the hybrids reaches 10^10^ A W^−1^ at 130 K and 5 × 10^8^ A W^−1^ at room temperature. The lifetime of excess electrons is so long as to cause a nearly persistent photoconductance within experimental timescales. A large positive gate voltage pulse can be applied to recover the current to the initial state. Because, positive gate voltage would induce concentrated electrons and accelerate the recombination of trapped holes, as discussed in Section 2.3.1. Upon this regime, an optoelectronic memory can be developed. Another similar hybrid structure based on graphene and MoS_2_ was achieved by Zhang et al.^88^ The photogain even exceeds 10^8^. In 2015, Liu et al. fabricated a carbon nanotube–graphene hybrid photodetector with detection spectrum ranging from 400 to 1550 nm.^39^ As shown in Figure 9c, the negative shift of *I*_ds_–*V*_g_ trace after illumination indicates that the photoexcited holes are left in the CNT film which modulates the conductance of graphene. The gain of this device is on the order of 10^5^ and the response time is about 100 µs, implying an outstanding calculated GBP of 10^9^ Hz. In the same year, Lee et al. attempted to incorporate graphene with another hot researched light absorption material—CH_3_NH_3_PbI_3_ perovskite layers, as shown in Figure 9d. However, the performance of this hybrid is not so satisfying compared to the mentioned structures above. Moreover, the valid detection range is limited in the visible wave band due to light absorption relying on the perovskite.

The performance of most reported hybrid devices based on graphene is deserved owing to its superiorities of high mobility and tunability. Another advantage of broadband absorption is usually ignored due to its thickness. Therefore, such devices have a response spectrum greatly dependent on the gating material. It is of great importance to realize a graphene photodetector with longer detection wavelength. In 2014, a sandwiched design of phototransistor consisting of a pair of stacked graphene monolayers separated by a thin Ta_2_O_5_ tunneling layer was achieved by Liu et al.^91^ As shown in Figure 9b, under illumination, photoexcited hot carriers generated in the top monolayer graphene tunnel into the bottom layer while holes remain in the top layer, resulting in a strong photogating effect on the channel conductance. Since the bottom graphene is lightly p‐doped, the concentrated holes would induce a negative photoconductance. This could also be concluded according to the negative *I*_ds_–*V*_g_ trace shift with increasing incident power or directly observed through the temporal photoresponse. A responsivity of higher than 1 A W^−1^ for NIR–MIR (1.3–3.2 µm) waveband was obtained at room temperature. This result addresses the key significance of photogating for 2D material‐based infrared photodetection.

## 3 General Photogating

For the photogating discussed in Section 2.3, no matter the photo‐FET consists of bare material or is a hybrid structure, the channel material directly involves the transport of at least one type of the photoexcited carriers. Large gain produced by long carrier lifetime inhibits the bandwidth. Here we define “general photogating” as a way of indirect photodetection controlled by photovoltage. Or in other words, the channel materials do not respond to the detected light itself and the gain is only produced by the photovoltage instead of a prolonged carrier lifetime. This type of photogating may achieve both high gain and high bandwidth. Here, we mainly introduce three cases of general photogating according to the reported results very recently.

### 3.1 Photovoltage FETs (PVFETs)

**Figure 10:**
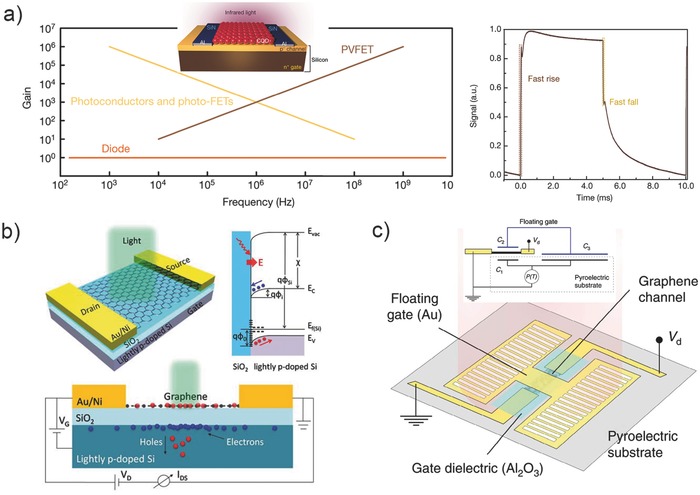
Three cases of general photogating. a) Left panel: the simulated relationship between gain and frequency for the PVFET, and photoconductors and photo‐FETs (inset: schematic diagram of the PVFET). Right panel: temporal response of the PVFET. Reproduced with permission.^40^ Copyright 2017, Nature Publishing Group. b) Graphene photodetector based on interfacial gating and its energy band explanation. Reproduced with permission.^44^ Copyright 2016, Optical Society of America. c) Schematic view and circuit diagram of an individual pyroelectric graphene bolometer. Reproduced with permission.^95^ Copyright 2017, Nature Publishing Group.

The concept of PVFETs was put first forward by Adinolfi and Sargent in their research on QD/silicon hybrid photodetectors reported in 2017.^40^ The channel of the device is lightly p‐doped silicon thin film epitaxially grown on heavily n‐doped silicon which acts as the gate (see the inset of left panel in **Figure** **10**a), and is deposited by a PbS QD layer with judiciously engineered heterointerface passivation. Illumination beyond the bandgap of silicon excites free e–h pairs in PbS QDs and generates a voltage at the interface which shrinks the depletion region in silicon channel. The gain can also be described using Equation (3). The model of junction FET was applied to investigate the relation between the cut‐off frequency and gain. Gain has been convinced to be proportional to the transconductance *g*_m_ according to Equation (3). Meanwhile, the cut‐off frequency determined by *g*_m_/2*πC*_TOT_ also shows a proportional dependence on the transconductance. Therefore, this type of phototransistor enables simultaneous high gain and high bandwidth, as depicted in Figure 10a (left panel). In this regime, a gain of 10^4^ and a bandwidth of 10^5^ Hz were achieved, leading to a high GBP of ≈10^9^ Hz. Further simulation even shows that a value up to 10^13^ Hz could be obtained in principle, which is higher than that of photodiodes, photo‐FETs, and photoconductors.

The performance of this PVFET greatly depends on the quality of the QD/silicon rectifying junction, which is essentially determined by the band alignment while greatly affected by the defects and interface states. Compared to the exfoliated 2D materials in QD/2D‐material hybrids,^31^, ^41^, ^42^, ^43^, ^84^, ^85^, ^86^, ^87^ the surface of MBE grown silicon should be easier to be cleaner. However, due to the imperfect quality of QDs and nonabsolutely impurity‐free spin coating, this PVFET still suffers a relatively long tail in the fall time of photocurrent (Figure 10a, right panel). A good rectifying output characteristic implies an effective electron barrier from QDs to silicon. This barrier impedes the injection of photogenerated electrons from QDs into silicon and results in a photovoltage with high operation efficiency. Though the similar level of GBP of this PVFET has also been achieved by other hybrids dominated by photogating, the dark current density is much lower owing to the gate controlled depletion of thin channel. If a merit is defined as *F* = GBP/*J*_D_^1/2^ where *J*_D_ is the dark current density, then the QD/silicon PVFET outperforms all the reported QD‐based detectors by at least one order of magnitude.

### 3.2 Interfacial Gating

Early in the year of 2006, Marcus et al. observed that the visible light absorption in the silicon substrate can generate a photovoltage to gate the nanotube device.^35^ They called this mechanism photogating. Photogating dominated photocurrent is significantly larger than that due to direct e–h separation, even though they used a heavily p‐doped silicon as the gate. Ten years later, Guo et al. used lightly p‐doped silicon substrate to gate monolayer graphene and achieved an outstanding result.^44^ As shown in Figure 10b, downward band bending at the Si/SiO_2_ interface is caused by the localized states such as positive charge states at the oxide–silicon interface, thus forming a built‐in electric field near the interface. Under illumination, photogenerated e–h pairs will be separated due to the built‐in field. Subsequently, holes will diffuse toward the bulk silicon whereas electrons accumulate at the interface. As a result, an additional negative gate voltage will be applied on the graphene channel. To obtain an effective gating effect, lightly p‐doped silicon is preferred. Because, heavily doped silicon has a much shorter lifetime of excess electrons. Another similar graphene/SiO_2_/lightly p‐doped Si device has been checked by another group and the same conclusion has been obtained.^92^ This monolayer graphene photodetector dominated by interfacial gating^44^ shows an outstanding performance. It enables a highly sensitive detection for a light signal of less than 1 nW and a fast response time of ≈400 ns. The GBP reaches as high as 10^9^ Hz.

The high mobility of graphene mainly contributes to the considerable net photocurrent. The transconductance of a graphene metal‐oxide‐semiconductor FET (MOSFET) in the nonsaturation region can be written as *g*_m_ = *μC*_ox_ *V*_ds_(*W*/*L*), where *C*_ox_ is the dielectric capacitance per unit area, *W* and *L* are the channel width and length of graphene, respectively. Hence *I*_ph_ = *μV*_ds_(*C*_ox_Δ*V*_g_)(*W*/*L*),^41^, ^44^ which can also be written as *I*_ph_ = Δ*Q*/τ_T_, where Δ*Q* is the photoinduced electric charge concentrated at the interface and τ_T_ is the carrier transit time in graphene channel. It is concluded that the net photocurrent is independent on the thickness of SiO_2_, while it can be greatly affected by the carrier transit time and the amount of concentrated charge induced by illumination. Because of the much lower mobility (0.1–10 cm^2^ V^−1^ s^−1^) of MoS_2_ than that of graphene, the MoS_2_ device for experimental control did not show obvious interfacial gating effect. Moreover, using Al_2_O_3_ instead of SiO_2_ resulted in a much weaker net photocurrent.^44^ Apart from the low carrier mobility, another possible reason why the MoS_2_ device for control did not show obvious interfacial gating could be that the MoS_2_ responses to the incident light itself which should result in a positive photoconductance, while the interfacial gating derived from SiO_2_/Si interface would only induce a negative gate photovoltage. These combined effects make it harder for MoS_2_ to exhibit an obvious interfacial gating.

Like conventional photogating, the photocurrent of interfacial gating varies with the gate voltage as described in Equation (2). However, the response speed may not be necessarily affected by the gate voltage. Because, gate voltage has nothing to do with the carrier lifetime and trap states in graphene. Hence, this type of photogating may allow both high gain and high bandwidth just as PVFET. Nevertheless, the gate voltage may change the band bending at interface, thus affecting Δ*V*_g_. Interfacial gating paves the way to explore novel high‐performance phototransistors consisting of suitable channel material and substrate.

### 3.3 Graphene‐Based Pyroelectric Bolometer

Thermal detectors for MIR detection are widely studied and applied owing to the no requirement for cooling. To date, 2D graphene‐based thermal detectors consist of photo‐thermoelectric detectors,^25^, ^26^, ^27^ bolometers,^93^, ^94^ pyroelectric detectors.^95^ Among them, graphene thermopile has been fabricated for imaging system^96^ whereas the temperature coefficient of resistance (TCR) of such bolometers is unsatisfying.^97^ Very recently, Sassi et al. integrated monolayer graphene with *z*‐cut LiNbO_3_ crystal and designed a graphene‐based pyroelectric bolometer with TCRs up to 900% K^−1^.^95^ The structure of an individual device is shown in Figure 10c (inset is the circuit diagram), where the conductance of monolayer graphene (coated by Al_2_O_3_) is modulated by a pyroelectric substrate and an Au floating gate. When irradiated by a heat source, the temperature change induced pyroelectric charge Δ*Q* on capacitor C_3_ depends on the area as Δ*Q* = *p*Δ*TA*_C3_, where *p* (µC m^−2^ K^−1^) is the pyroelectric coefficient, Δ*T* is the temperature change of substrate, and *A*_C3_ is the area of capacitor C_3_. This accumulating charge can entirely be provided by capacitor C_2_ due to the charge conservation, thereby an induced top‐gate voltage Δ*V*_g_ is generated to modulate the conductance of graphene, which can be written as Δ*V*_g_ = Δ*Q*/C_2_ = *p*Δ*TA*_C3_/(ε_0_ε_r_ *A*_C2_/*t*), where ε_0_, ε_r_, *A*_C2_, and *t* are the vacuum permittivity, the relative permittivity of the oxide, the area of capacitor C_2_, and the oxide thickness. Since the current change induced by Δ*V*_g_ is depicted as Equation (2), the TCR (Δ*I*/*I*_0_) greatly depends on the geometrical ratio *A*_C3_/*A*_C2_. By enlarging the device pixel to 300 × 300 µm^2^ (*A*_C3_/*A*_C2_ ≈ 66), a TCR as high as 600% K^−1^ was obtained, which made it sensitive to the human hand at a distance of ≈15 cm. Moreover, this device can be operated in direct current because of the absence of leakage current, thereby there is no need for chopping. Though the authors did not even mention a word of photogating in their paper, the thought of using photovoltage to modulate the channel conductance is common. This type of indirect detection pushes the performance of hybrid detectors into a higher level.

## 4 Summary and Perspectives

In this review, photogating in various LDPDs has been discussed. As a special photoconductive effect, we first introduce the photoconductive gain and point out the very different expression of gain of photogating as Equation (3), which is in agreement with the definition. Subsequently, we have discussed the main possible origins and structures of photogating and summarized the behaviors of photogating in LDPDs. The characteristics include competing gain and bandwidth, nonlinear power dependence of gain and responsivity, horizontal *I*_ds_–*V*_g_ trace shift, and unexpected NPC. Among them, the NPC is very special because it is hardly observed in traditional thin‐film photodetectors. Moreover, if a NPC was found in hybrid structures, we could almost confirm the existence of photogating. Then, the recent progress on photogating in different low dimensional systems has been introduced, including NWs, 2D materials, hybrid QD/2D material structures, and graphene‐based hybrids. Furthermore, we have discussed three cases of general photogating according to the reported studies very recently. General photogating has no competing gain and bandwidth, which paves the way to design hybrid photodetectors with more excellent performance.

**Figure 11:**
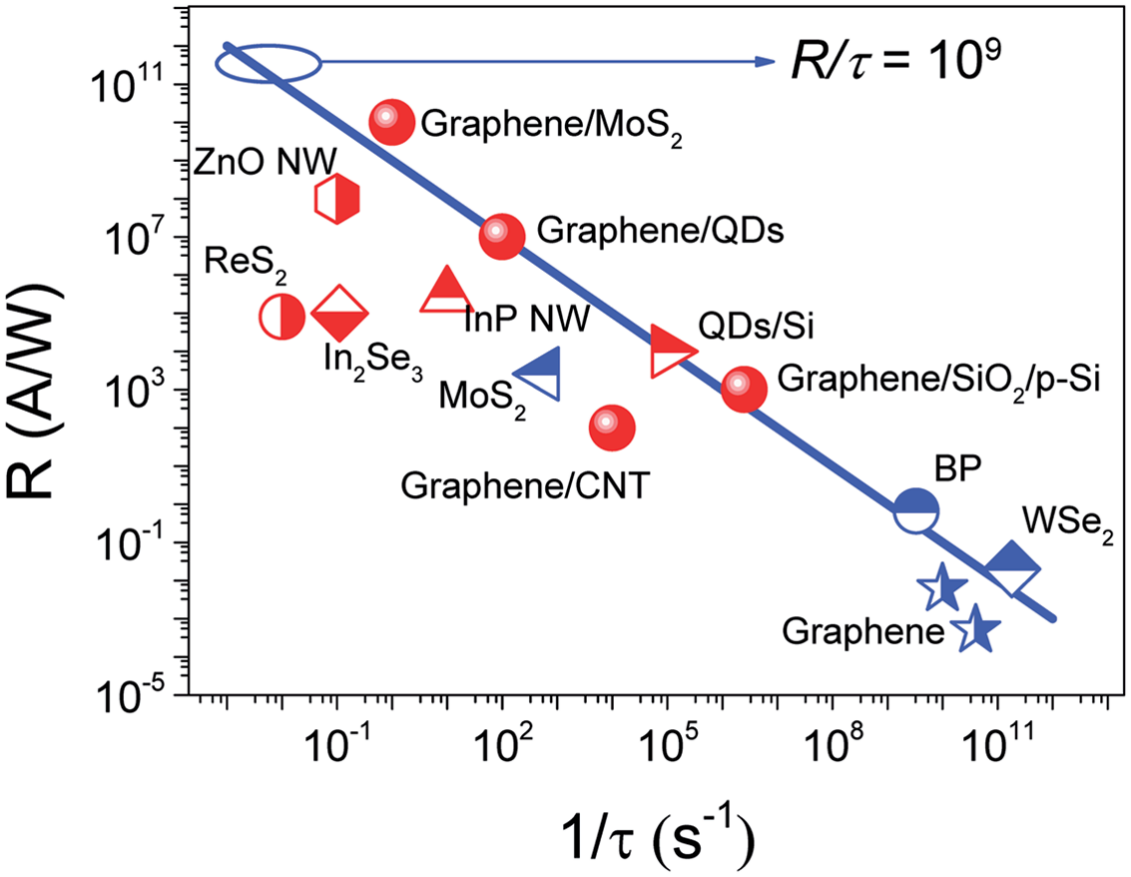
Responsivity and response time of part current state‐of‐the‐art low dimensional photodetectors.^17^, ^31^, ^32^, ^39^, ^40^, ^44^, ^47^, ^67^, ^99^, ^100^, ^101^, ^102^, ^103^, ^104^ The blue line represents a typical magnitude order of GBP for traditional high‐performance thin‐film photodetectors. Red symbol indicates that this is a photogating enhanced photodetector.

Photogating deserves tremendous attention, because not only it is widely found in LDPDs but also the related photodetectors show remarkable detection ability, especially for weak light. Photogating directly means high responsivity as discussed in the above sections (see Table 2). Moreover, a large GBP could also be achieved by photogating. We summarize part of the current state‐of‐the‐art LDPDs with high GBP, as shown in **Figure** **11**. It is self‐evident that photogating plays a crucial role for improving LDPDs to be comparable with traditional thin‐film photodetectors with GBP as high as 10^9^. Now, we come to the issue that how to evaluate a LDPD. First of all, the performance of a LDPD itself should be truly reflected. For instance, the responsivity and response time should be measured at the same gate voltage and incident light power density. Because, these values could vary so much at different conditions of gate voltage or light power. Due to the special practical application, a trade‐off has to be made between gain and bandwidth. Graphene‐based hybrid LDPDs prefer to show an ultrahigh gain owing to the high mobility. However, the dark current is not suppressed as a result of the lack of bandgap, thus greatly restricting the signal‐to‐noise ratio. We recommend to use the ratio between the GBP and the square of dark current (*F* = GBP/*J*_D_^1/2^) to evaluate the whole performance of a photogating dominated LDPD, as put forward by Adinolfi and Sargent.^40^

Photogating can help to design novel low dimensional IR photodetectors with high performance. As discussed in this review, the shallow trap states dominated room‐temperature MIR BP photodetector^29^ has a higher gain than low temperature IR photodiodes and a faster response speed than room‐temperature thermal detectors like vanadium oxide and amorphous silicon. InAs NWs with abundant surface states show little response to MIR light, while taking advantage of the NW depletion induced by photogating can achieve a sensitive detection for MIR light.^75^ Incorporating the sufficient light absorption of PbS QDs with the high mobility of graphene results in a highly sensitive photodetector with broader detection spectrum ranging from visible to NIR.^31^ And, photogating in sandwiched graphene/Ta_2_O_5_/graphene even enables graphene photodetector to realize a MIR light detection at room temperature with considerable responsivity.^91^ Such examples have demonstrated the availability of photogating in novel low dimensional IR photodetectors. However, there are still some challenges. For photogating induced by trap states, it is hard to control the number and type of trap states by means of material‐growth technology or exfoliation method of 2D materials. This leads to devices with less reproducibility. Many hybrid structures use graphene as the device channel. This leads to high gain but large dark current. Hence, the hybrid QD/graphene image sensor array needs an external row of blind pixels that are used to subtract the dark signal.^87^ Moreover, due to the nonuniformity of spin coating of QDs and uncertain distribution of detects and trap states, the pixel drift and spread in sensitivity are too large to make this image array succeed to obtain extreme weak‐light images at current stage, despite the ultrahigh gain. In addition, this type of photodetectors with relatively low bandwidth fails to meet the requirement of critical applications. However, this could be overcome by means of general photogating, where dramatic improvement of performance could be achieved. Photovoltage FETs have high bandwidth, low dark current, and extended detection range, which outperform black silicon.^40^ Interfacial gating enhanced graphene photodetector shows much higher responsivity than pure graphene.^44^ Because the only connection between the device channel and the substrate is the photovoltage produced at the gate, this kind of photodetector allows multiple combinations of suitable channel materials and substrates for special applications, including novel room‐temperature high‐performance IR photodetectors.

## Conflict of Interest

The authors declare no conflict of interest.
